# Assessment of Risk Factors for Acute Kidney Injury with Machine Learning Tools in Children Undergoing Hematopoietic Stem Cell Transplantation

**DOI:** 10.3390/jcm13082266

**Published:** 2024-04-13

**Authors:** Kinga Musiał, Jakub Stojanowski, Monika Augustynowicz, Izabella Miśkiewicz-Migoń, Krzysztof Kałwak, Marek Ussowicz

**Affiliations:** 1 Department of Pediatric Nephrology, Wrocław Medical University, Borowska 213, 50-556 Wrocław, Poland; 2 Department of Nephrology and Transplantation Medicine, Wrocław Medical University, 50-556 Wrocław, Poland; jakub.stojanowski@student.umw.edu.pl; 3 Clinic of Pediatric Nephrology, University Clinical Hospital, Borowska 213, 50-556 Wroclaw, Poland; 4 Clinical Department of Pediatric Oncology and Hematology, Mother and Child Health Center, Karol Marcinkowski University Hospital, 65-046 Zielona Góra, Poland; 5 Department of Pediatric Bone Marrow Transplantation, Oncology and Hematology, Wrocław Medical University, 50-556 Wrocław, Poland; krzysztof.kalwak@umw.edu.pl (K.K.); ussowicz@o2.pl (M.U.)

**Keywords:** acute graft versus host disease, acute kidney disease, artificial intelligence, random forest classifier, tubular damage

## Abstract

**Background**: Although acute kidney injury (AKI) is a common complication in patients undergoing hematopoietic stem cell transplantation (HSCT), its prophylaxis remains a clinical challenge. Attempts at prevention or early diagnosis focus on various methods for the identification of factors influencing the incidence of AKI. Our aim was to test the artificial intelligence (AI) potential in the construction of a model defining parameters predicting AKI development. **Methods**: The analysis covered the clinical data of children followed up for 6 months after HSCT. Kidney function was assessed before conditioning therapy, 24 h after HSCT, 1, 2, 3, 4, and 8 weeks after transplantation, and, finally, 3 and 6 months post-transplant. The type of donor, conditioning protocol, and complications were incorporated into the model. **Results**: A random forest classifier (RFC) labeled the 93 patients according to presence or absence of AKI. The RFC model revealed that the values of the estimated glomerular filtration rate (eGFR) before and just after HSCT, as well as methotrexate use, acute graft versus host disease (GvHD), and viral infection occurrence, were the major determinants of AKI incidence within the 6-month post-transplant observation period. **Conclusions**: Artificial intelligence seems a promising tool in predicting the potential risk of developing AKI, even before HSCT or just after the procedure.

## 1. Introduction

Acute kidney injury (AKI) is a common complication affecting up to 84% of children undergoing hematopoietic stem cell transplantation (HSCT) [1,2,3,4]. A recent meta-analysis has established the overall pooled incidence of pediatric AKI after HSCT to be 47% [5]. The scale of this complication necessitates the constant improvement of procedures mitigating, or at least diminishing, the frequency of AKI occurrence in this population [1]. The list of potential risk factors for AKI after pediatric HSCT is still incomplete. According to a recent analysis, the main factors are a matched unrelated donor, cord blood transplantation, and sinusoidal obstruction syndrome [5]. Other studies confirm the influence of unrelated donors, underlining the additional roles of viral infections, malignant disease as the indication, drug nephrotoxicity, complications like thrombotic microangiopathy, or an older child’s age, in aggravating the risk of AKI development after HSCT [6,7,8,9,10]. Recent data also point to the impact of initially increased estimated glomerular filtration rate (eGFR) values on further AKI recurrence [11].

Artificial intelligence (AI) tools have already been used to assess the risk factors for AKI development in specific groups of patients, including those after cardiosurgery or on intensive care units [12,13,14]. AI implementation in the analysis of pediatric AKI has been highly successful in the neonatal population, but has not covered the issue of post-HSCT AKI sufficiently [15]. Our preliminary results suggested the potential of AI when predicting incipient renal dysfunction in children undergoing HSCT, with the use of damage markers [16].

Therefore, our current aim was to analyze the classical risk factors for AKI in the population of children undergoing HSCT, with the use of a machine learning methodology. Our goal was to create an AI model with sufficient positive and negative predictive power to assess the risk of AKI incidence in the pediatric population within the first 6 months after HSCT.

## 2. Materials and Methods

### 2.1. Patient Characteristics

This retrospective analysis centered on the medical records of 173 children who underwent their first allogeneic HSCT in the years 2016–2018 in the Department of Pediatric Bone Marrow Transplantation, Oncology and Hematology. The patients were observed from the introduction of conditioning therapy. Then, follow-up lasted for 6 months and covered the early post-HSCT period (24 h after HSCT and then 1, 2, 3, and 4 weeks after transplantation), the intermediate interval (8 weeks and 3 months post-HSCT), and the late observation period (6 months after HSCT).

The exclusion criteria for HSCT children were an age over 18 years or below 2 years (in the latter case, owing to disproportionate eGFR values, which are significantly lower compared to those of older children). In total, 135 children (57 girls and 78 boys, with a mean age of 8.27 ± 5.14 years) met the above-mentioned criteria.

Conditioning therapy was based on a myeloablative (busulfan- or treosulfan-based, with the addition of any of the following: cyclophosphamide, fludarabine, or thiotepa) or non-myeloablative (cyclophosphamide, fludarabine) regimen. The patients followed the prophylaxis against graft versus host disease (GvHD), consisting of the pre-transplant anti-thymocyte globulin (ATG), cyclosporine A given from the day preceding transplantation, and three methotrexate doses provided on the 1st, 3rd, and 6th days post-HSCT.

### 2.2. Serum Creatinine and eGFR Values

The frequency of kidney function evaluation at fixed time points relied on hematological protocols. The serum creatinine concentration was measured in a sequential way: before conditioning, at 24 h after HSCT, 1, 2, 3, 4, and 8 weeks after HSCT, and then 3 and 6 months after HSCT. The eGFR values were calculated based on the Schwartz formula [17]. The eGFR current values were compared to the pre-transplantation ones.

### 2.3. AKI Diagnosis

AKI diagnosis was made based on the pRIFLE criteria, assessing the degree of eGFR decrease, and KDIGO classification, evaluating the serum creatinine rise [18]. The urine output and fluid overload criteria could not be assessed due to unavailability of the retrospective data. Hyperfiltration was defined as eGFR ≥ 140 mL/min/1.73 m^2^, based on the pediatric experience and data from a meta-analysis [19,20].

### 2.4. Classical Statistical Analysis

Continuous variables were presented as mean ± standard deviation (SD), while categorical variables were shown as frequencies and percentages. The continuous variables were compared by the means of ANOVA and Student’s *t*-test. Correlations between categorical variables were identified with the use of chi-square or Fisher’s exact tests. A *p*-value < 0.05 was considered significant. Calculations were performed with the use of TIBCO Statistica v.13.3 (TIBCO Software, Inc., Palo Alto, CA, USA).

### 2.5. Machine Learning Methods

#### 2.5.1. Machine Learning Is a Domain of Artificial Intelligence Aimed at Imitating the Decision-Making Process Carried out by Humans

The choice of random forests as the preferred method of analysis was justified by the simplicity of data preparation, lack of need to scale them, and transparency in model analysis.

#### 2.5.2. Model Performance Measures Are Classically Described as the Ratio of True Matches to Both Domains: Positives and Negatives

Precision is the machine learning equivalent of positive predictive power and is expressed as the ratio of true positive observations to all positive observations. In this way, it determines the chance that a positive value is true. Recall is the probability of a positive test result, depending on whether the observation is actually positive. In statistics, recall is called sensitivity. The F1-score is the harmonic mean of precision and recall. The Matthews correlation coefficient (MCC) is an indicator converging to 1.0 as the overall model performance improves in all four fields of the confusion matrix [21]. The more true classifications there are and the fewer false ones, the higher the MCC value that the model achieves [21]. The MCC design provides insensitivity to set imbalances. This is particularly important when there are more representatives of the selected class in the validation set. Then, even an ineffective classification model can achieve high accuracy due to typing a larger group of labels.

The random forest classifier can be used to select input data for other machine learning models due to its fast model building [22]. A random forest requires less computational complexity, allows for searching the probability space of many possible solutions, and offers a visual form that is acceptable for interpretation. Neural networks require more data than random forests to converge to an optimal solution. In our work, we focused on discovering the predictors of acute kidney injury in patients after HSCT. Scientific publications confirm the particular effectiveness of RFCs in selecting critically important parameters for endpoint prediction [23,24].

#### 2.5.3. Selection of Input Data and Development of the Model

**Figure 1:**
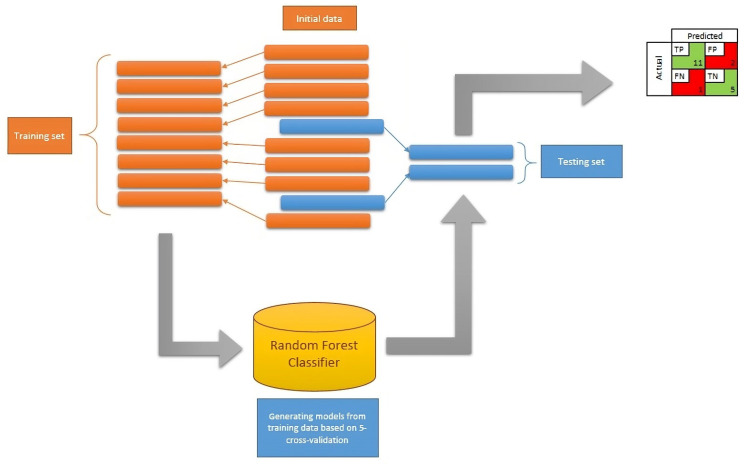
A graphical representation of the methodology for generating a random forest model. The input set is divided in a ratio of 80:20 into a training and testing set. The training set allows for generating the optimal set of input data needed for effective prediction. The testing set is used to simulate new patient data beyond the data originally available for training.

The original database was tested for the completeness of timely observations, covering the period from the first day before conditioning to the sixth month after HSCT, and thus, finally, 93 patients were included. The final database was divided into training and testing sets in a ratio of 80:20 [25]. Random forest models were created on the training data, the best of which was validated on the testing set. The model input data were selected by applying the brute force method and checking all combinations of input parameters. The subsets were obtained by recursively calling the random forest classifier generating function. The obtained model was evaluated based on 5-cross validation against MCC, and the results were saved in an external file. The model with the highest score was then selected and records from the testing set were used as inputs (Figure 1).

#### 2.5.4. Feature Importance

The input is an unclassified set of data associated with labels. The purpose of the classification model is to divide this set in such a way as to obtain possibly uniform subsets of data. An ordered arrangement of such divisions, described in numerical terms, is represented by a decision tree. A set of such decision trees, built on the basis of random divisions, is a random forest classifier. Feature importance tells us what contribution a specific variable makes to this classification of input data. The higher the value, the more crucial the feature [26].

Constructing a decision tree uses a minority or equality relationship, when formulating conditions at partition nodes. Therefore, the random forest classifier does not distinguish between discrete and continuous variables, or between quantitative or qualitative variables. It is important to prepare the database in such a way that the data placed on the number line are ordinal. So, as the numerical value increases, there is a relative change in the described phenomenon. For this reason, categorical variables should retain the properties of numerical order.

## 3. Results

### 3.1. Clinical Data Concerning the HSCT Patients

Detailed demographic and clinical data are shown in Table 1.

### 3.2. Serum Creatinine and eGFR Values

Serum creatinine concentrations before alloHSCT were within the normal range in all patients. They decreased significantly 24 h post-transplant and remained lower until the first week after HSCT. The return of the serum creatinine concentration to pre-transplantation values occurred after 3 weeks. Then, the values started increasing from the fourth week after transplantation and continued rising until the sixth month post-HSCT (Table 2).

In none of the patients was eGFR < 60 mL/min/1.73 m^2^ before HSCT. In the vast majority of cases (92%), eGFR values exceeded 90 mL/min/1.73 m^2^. Forty percent of patients had hyperfiltration before the procedure, and after HSCT, this share increased to 63%. The highest eGFR values were observed 24 h and 1 week after HSCT. The eGFR records returned to the values observed before HSCT only after 3 weeks (Table 2). Subsequently, the mean eGFR values continued diminishing from the fourth week post-transplant and did so until 6 months after HSCT (Table 2).

### 3.3. The Incidence of AKI

During the entire time of follow-up, the features of AKI, according to the pRIFLE criteria, were noted in 54% of the patients. A fall in eGFR value > 25% (risk or stage 1) occurred in 58 patients, whereas a 50% eGFR decrease (injury or stage 2) was seen in 14 patients (Table 2). None of the patients experienced failure, i.e., a decrease in eGFR by 75% (stage 3).

Only 26% of the children fulfilled the criteria of AKI according to the KDIGO guidelines. Twenty-seven children presented with AKI stage 1, whereas nine patients were diagnosed with stage 2.

### 3.4. Preparing the Dataset to Build the Model

The data for the random forest classifier did not require scaling or normalization. The data used to construct the model were complete.

Building the model required dividing the database in an 80:20 ratio into two subsets, one for preparing the model through training, and the other for verifying the model’s predictive ability on new data [25]. The data from the testing set were completely new for the model. Model building was based on selecting the top model using five-cross validation on the training set (Table 3).

The way to construct a random tree is to use conditions written using minority or equality relations. Therefore, no special preparation of variables is required before implementing modeling.

### 3.5. Model Predicting AKI Incidence during the Observation Period

**Figure 2:**
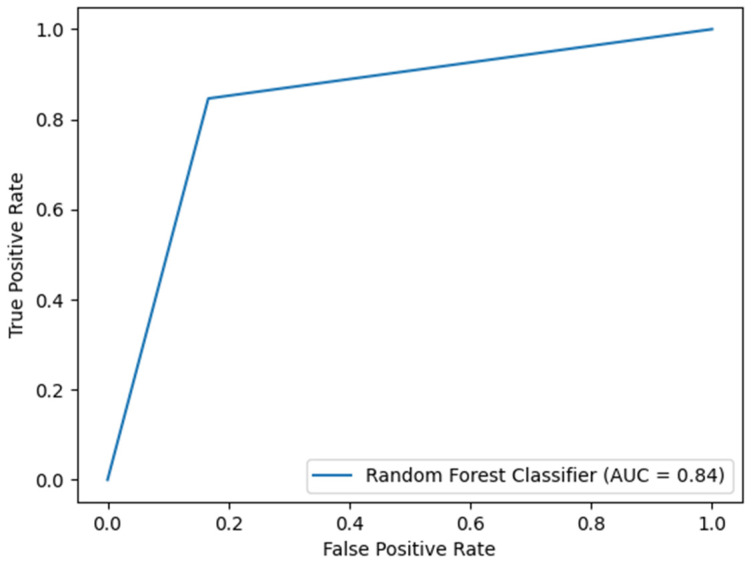
The satisfactory discriminatory ability of the developed model allows for its practical application. The area under the ROC curve was 0.8397, with corresponding lower and upper limits of the confidence interval (CI) of 0.6588 and 1.0000, respectively.

Based on patient data, a model was built using the random forest method. Such a model is based on a set of decision trees that classify the input data into one of the labels. For this study, these were the absence of AKI or the presence of AKI during the follow-up period. The random forest model correctly classified 84.21% of the records from the test set. It showed a precision (positive predictive ability) and sensitivity of 0.8528 and 0.8421, respectively. The MCC value of 0.6548 gave a satisfactory predictive value, with the potential for further improvement. The discriminatory ability was also at a significant level, and so was the area under the ROC (0.8397). The lower and upper limits of the confidence interval (CI) for the area under the receiver–operator curve were 0.6588 and 1.0000, respectively (Figure 2).

The confusion matrix in Table 3 reflects the full characteristics of the classification of patients from the test group into appropriate categories in the model developed on the basis of the training data, which constituted 80% of the original data.

The positive predictive value of the absence of acute kidney injury was 0.71, the sensitivity was 0.83, and the F1-score value was 0.77. The positive predictive power of acute kidney injury during the follow-up period was 0.92, the sensitivity was 0.85, and the F1-score was 0.88. Hence, it could be inferred that this model had an improved ability to detect acute kidney injury. The feature importance for the input parameters is presented in Table 4.

**Figure 3:**
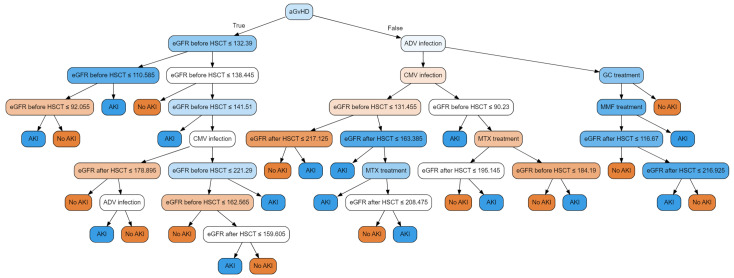
The variables: eGFR before HSCT and eGFR after HSCT appear significantly more often than average in the above tree. A random forest consists of many similar trees. In the case of the model in question, there are 17 of them. A single tree allows for selecting one predicted endpoint. Several trees cast their votes and the final result is chosen by majority rule.

The significance of the variables can be seen in an example decision tree, which is a part of the random forest classifier (Figure 3). The statistics presented in Table 4 apply to the entire model, but we can observe at what stage a given condition defines the division of the input set. The occurrence of acute GvHD divides the data in the first stage, but they are more often divided based on the values of eGFR before HSCT and eGFR after HSCT. When eliminating outliers, the complexity of decision trees can be reduced. Some divisions lead to the clarification of individual values and do not contribute much to the overall classification. In the current study, similar data manipulations were omitted.

## 4. Discussion

AKI is one of the most common complications in the course of the HSCT procedure and during the post-transplantation period. The background of kidney dysfunction in patients after HSCT is multifactorial [27]. AKI may result from pre-renal, renal, or post-renal etiologies, although simultaneity of various pathological mechanisms is more the rule than the exception [28,29]. Moreover, a cumulative impact of medications given during conditioning, engraftment, as well as prevention of complications such as GvHD or infections make the distinction of a single factor’s influence impossible. The existence of overlapping mechanisms necessitates the creation of models able to validate a single variable’s importance in confrontation with other factors, and AI fulfills these conditions [30,31].

Therefore, we identified the factors associated with the occurrence of pediatric AKI until 6 months after transplantation, with the use of random forest models. This allowed the identification of key features predicting the AKI incidence. One of the strengths of this approach is that it uses only two quantitative variables, routinely assessed during the procedure of HSCT: eGFR before conditioning therapy, and eGFR just after HSCT.

On the other hand, it was surprising that eGFR values turned out to be the major determinants of kidney function in the population affected by sarcopenia and metabolic disturbances. The above-mentioned anomalies, both altering serum creatinine values and subsequently aggravating hyperfiltration, justify such assessment, although imperfect, among the hematological protocols of kidney function evaluation [32,33,34]. The concomitant existence of two eGFR values, before and just after HSCT, in one model indirectly confirms previous observations of high eGFR values’ influence on AKI recurrence in HSCT children [11]. It also suggests the significance of a preserved renal functional reserve (RFR), represented here by the most prominent increase in eGFR value 24 h after HSCT versus the pre-transplantation record, for the prediction of AKI incidence [35,36].

Indeed, discussion of the impact of the RFR on recovery from AKI is not new, and we now have a broad perspective on RFR’s usefulness across clinical nephrology [37]. However, technical challenges concerning the methods of GFR stimulation and its further adequate measurement under dynamic conditions require standardization [38]. Consequently, prospective adjustment of the currently available methodology for pediatric specificity is needed, though it appears challenging. However, the first promising results correlate an RFR > 20% before HSCT with a subsequent recovery from AKI after transplantation [39]. Therefore, larger studies, aimed at defining RFR thresholds in children planned for HSCT, or at finding surrogate markers for such assessment, are awaited.

Proteinuria seems another candidate quantitative variable for the evaluation of AKI risk. Surprisingly, though one of the most evident predictors of chronic kidney disease progression, this has not attracted comparable attention regarding its impact on acute kidney damage. A meta-analysis published in 2015 established the role of an increased albumin-to-creatinine ratio (ACR) as a strong risk factor for AKI development, whereas the level of pre-AKI proteinuria conditioned renal recovery or non-recovery in patients requiring dialysis [40,41]. Similarly, according to a recent systematic review, pre-operative proteinuria was connected with a greater risk of post-operative AKI in adults undergoing radical or partial nephrectomy due to renal cancer [42]. However, all those studies concerned adults with proteinuria accompanied by decreased eGFR, as opposed to our pediatric post-HSCT population with hyperfiltration. Moreover, routine urinalysis in HSCT children was not accompanied by urine creatinine evaluation, so ACR values were not available.

On the contrary, our prospective study on the role of novel damage markers, such as NGAL, KIM-1, and IL-18, in AKI assessment in HSCT children gave satisfactory results regarding the prediction of incipient kidney dysfunction 4 weeks after transplantation [16]. Unfortunately, we could not verify these results on the current database, owing to its retrospective character.

Apart from the quantitative variables found to be significant for our RFC model, the important qualitative variables were as follows: essential elements of the therapeutic regimen, complication incidence, and random events related to infections. This proved how complex the interrelations can be between various destructive factors acting during HSCT.

In our model, aGvHD, methotrexate use, and CMV/ADV infection were qualitative variables of known impact on AKI incidence. According to the literature, the use of conditioning regimens is one of the established AKI risk factors [2,43,44]. Moreover, the cumulative toxicity of busulfan, cyclophosphamide, cytarabine, melphalan, thiotepa, and total body irradiation may amplify a negative effect.

However, the list of potentially nephrotoxic drugs, requiring dose adjustment to the level of kidney dysfunction, contains methotrexate, calcineurin inhibitors, cyclophosphamide, antibiotics, and anti-fungal and anti-viral drugs [45]. Methotrexate shows direct toxicity towards renal tubules, whereas calcineurin inhibitors act through multiple unfavorable mechanisms, including endothelial damage by increased oxidative stress, arteriolar vasoconstriction due to suppression of vasodilators and vasoconstrictor predominance, as well as thrombotic microangiopathy [46].

Moreover, the renal mechanism of AKI is usually accompanied by pre-renal azotemia in the course of dehydration. The latter results from general side-effects of chemotherapy such as vomiting, diarrhea, or insufficient fluid intake because of mucositis [43]. When added to the negative caloric balance and cachexia, increased generation of uremic toxins is inevitable.

Acute GvHD, identified by our model as a factor associated with AKI at 6 months after HSCT, is per se an independent risk factor for kidney injury [2,46], and our model has confirmed its significance in the pediatric HSCT population. The paradox of this situation is that prophylaxis against GvHD also carries the risk of triggering AKI, or at least of increasing its occurrence. Acute GvHD also turns out to be a risk factor of developing acute kidney disease (AKD), which is another unfavorable outcome in the long-term observation until 6 months after HSCT [47].

Recent data have also confirmed that the incidence of AKI is significantly higher in children with documented viral infections [9]. These results are in concert with our data deciphering the impact of ADV/CMV infections on AKI incidence.

Our study has limitations. The number of patients with complete available data was low and the time of observation was relatively short. Furthermore, certain data, such as 24 h diuresis, fluid overload, protein-to-creatinine ratio, serum cystatin C, or damage markers, were unavailable owing to either incongruity to hematological protocols or the retrospective character of the analysis. Nonetheless, due to the limited size of the database, numerous statistics were used to confirm the quality of the model.

Based on the collected data on children undergoing HSCT, it was possible to build a predictive model assessing acute kidney injury’s incidence, according to the pRIFLE criteria, within 6 months after the procedure. Such predictions can be made based on the information about the use of various medications or the history of infection. However, the key parameters turned out to be the glomerular filtration rate before the procedure and on the first day after the procedure.

Future testing on the growing number of variables and observations in the database may improve the model performance. Furthermore, artificial intelligence tools can help identify risk factors leading to AKI in HSCT patients. In this case, the input parameters used in the model were those determined before or shortly after transplantation, which allows for more effective early risk management. It is hereby possible to identify the patients at particular risk of AKI, with satisfactory quality of the presented random forest classifiers.

## 5. Conclusions

Generating an optimal random forest model allowed us to determine which features are associated with kidney damage and which input data are most important in the prediction of AKI. The kidney function before HSCT and just after the procedure turned out to be the strongest predictors of AKI in the 6-month post-transplant period. Other major AKI risk factors were previous chemotherapy and viral infections. AI tools revealed their potential in identifying patients at risk of AKI development before HSCT, giving way to personalized treatment and effective prophylaxis.

The tested random forest model allows for effective classification of pediatric patients, according to the risk of AKI occurrence within 6 months after HSCT. The presented solution is scalable and can be easily expanded, thus enabling improvement and driving for perfection with the growing amount of data.

## Figures and Tables

**Table 1:** Basic demographic and clinical data of 135 patients undergoing hematopoietic stem cell transplantation (HSCT).

Patient Characteristics	Number of Children	Percentage
Boys/girls	78/57	58/42
^1^ eGFR < 90 mL/min/1.73 m^2^	11	8
eGFR > 140 mL/min/1.73 m^2^	55	40
Unrelated donors	98	72
Matching 10/10	86	63
Peripheral blood stem cells	114	84
*Conditioning therapy*		
Fludarabine	119	88
Thiotepa	86	63
Treosulfan	69	51
Cyclophosphamide	31	22
^2^ *GvHD prophylaxis*		
Cyclosporin A	132	98
Anti-thymoglobulin	108	80
Methotrexate	105	77
Mycophenolate mofetil	20	15
*Infectious complications*		
BK virus	86	63
Cytomegalovirus	47	35
Adenovirus	38	28
Epstein–Barr virus	35	26
Bacterial	23	17
Fungal	2	1
Acute GvHD	77	57
Chronic GvHD	21	15

^1^ eGFR—estimated glomerular filtration rate; ^2^ GvHD—graft versus host disease.

**Table 2:** Kidney function and AKI occurrence according to pRIFLE criteria in the studied group in subsequent time points before and after HSCT.

Time Point	Serum Creatinine [mg/dL] Mean Value ± SD	eGFR [ml/min/1.73 m^2^] Mean Value ± SD	Risk Incidence [Number of Patients/%]	Injury Incidence [Number of Patients/%]
Before HSCT	0.58 ± 0.19	141 ± 44	0	0
24 h after HSCT	0.49 ± 0.15 ^a^	164 ± 51 ^b^	1/0.7	2/1.4
1 week after HSCT	0.49 ± 0.17 ^a^	166 ± 55 ^b^	1/0.7	0
2 weeks after HSCT	0.53 ± 0.18 ^a^	156 ± 55 ^b^	11/8	1/0.7
3 weeks after HSCT	0.58 ± 0.17	141 ± 50	16/12	1/0.7
4 weeks after HSCT	0.61 ± 0.18 ^a^	133 ± 43 ^b^	25/18	0
8 weeks after HSCT	0.69 ± 0.30 ^a^	123 ± 42 ^b^	39/29	3/2
3 months after HSCT	0.69 ± 0.26 ^a^	122 ± 41 ^b^	29/21	7/5
6 months after HSCT	0.64 ± 0.18 ^a^	127 ± 38 ^b^	23/17	0

AKI—acute kidney injury; HSCT—hematopoietic stem cell transplantation; SD—standard deviation; eGFR—estimated glomerular filtration rate; ^a^ *p* < 0.05 vs. serum creatinine before HSCT; ^b^ *p* < 0.05 vs. eGFR before HSCT.

**Table 3:** Confusion matrix for the presented model. TP = true positives, FP = false positives, FN = false negatives, TN = true negatives.

	Predicted			
Actual	TP		FP	
		11		2
	FN		TN	
		1		5

**Table 4:** The best random forest model used two quantitative variables and six qualitative variables.

Feature	Feature Importance
eGFR after HSCT ^b^	37.04%
eGFR before HSCT ^b^	35.78%
Methotrexate ^a^	8.54%
Cytomegalovirus ^a^	5.99%
Adenovirus ^a^	4.99%
Acute GvHD ^a^	4.04%
Mycophenolate mofetil ^a^	2.63%
Glucocorticoids ^a^	0.98%

^a^ qualitative variables, ^b^ quantitative variables.

## Data Availability

The datasets generated and analyzed during the current study are available from the corresponding author on reasonable request.
